# The Story of the Insane from Year to Year

**Published:** 1901-06-08

**Authors:** 


### THE STORY OF THE INSANE FROM YEAR TO YEAR.

(Thirteenth Series.)

### MONMOUTHSHIRE ASYLUM AT ABERGAVENNY.

This asylum contained 1,050 patients on January 1st, 1899, and exactly the same number on December 31st of the same year. There were 176 first admissions, 40 re-admissions, 69 recoveries, 12 discharged relieved, and 126 deaths. The average number resident during the year was 1,057, and the total number of persons under care was 1,255. Calculated on the admissions the percentage of recoveries was 31·9, and calculated on the average number resident the percentage of deaths was 11·9. The former percentage is lower than the Monmouthshire average, and the latter is more than 2 per cent. higher than the average. Of the year's deaths 55 were caused by cerebral and spinal diseases, 15 by consumption, 5 by pneumonia, 10 by bronchitis, and 3 by pleurisy. The assigned causes of insanity in 13 of the admissions were domestic trouble, adverse circumstances, &c., in 20 the cause was intemperance in drink, in 46 it was hereditary influence, and in 44 "previous attacks." Dr. Glendinning points out that 87 per cent. of the recoveries takes place in patients who have been less than a year in the asylum, and he explains the low recovery rate by the debilitated condition of the patients admitted in 1899. Indeed 40 of these admissions died within the year and 31 within six months. On the other hand he mentions the case of a male patient who recovered after 13 years' residence. The mortality from consumption was 1·2 per cent. of the total number resident. Dr. Glendinning thinks some of the cases may have been owing to the overcrowding of the asylum in 1898. "The committee have much pleasure in again bearing testimony to the very able and highly satisfactory manner in which the important and responsible duties have been discharged by Dr. Glendinning and the whole staff." The average cost per head per week was 7s. 4d.

### HEREFORDSHIRE COUNTY ASYLUM, ST. ALBANS.

This is the first annual report of the new asylum which was opened on April 7, 1899. There were during the year 122 first admissions, 5 re-admissions, and 2 deaths. Dr. Boycott, the medical superintendent, gives us a very interesting historical summary of the events which led to the building of the asylum, and he also gives a description of the asylum itself from which we learn that it contains 576 beds, with administrative department large enough for 800. Every ward, we are told, is complete in itself, containing dayrooms, dormitories, ward scullery, bootroom, storeroom, and sanitary block with lavatory, w.c.'s, and bath-room. In this list we miss the dining-room, and so we infer that the patients have their meals in the wards, or worse still, that they are taken three times daily to a general dining-room. It is painful to notice how often asylum architects commit this great mistake. The estate at Hill End is of the extent of 184 acres, and the buildings cover about eight acres of this. The period since the opening of the asylum is too short to make it desirable to quote any of the usual statistics.

